# Association between the number of fungiform papillae on the tip of the tongue and sensory taste perception in children

**DOI:** 10.1080/16546628.2017.1348865

**Published:** 2017-07-26

**Authors:** Hannah Jilani, Wohlfgang Ahrens, Kirsten Buchecker, Paola Russo, Antje Hebestreit, on behalf of the IDEFICS consortium

**Affiliations:** ^a^ Leibniz Institute for Prevention Research and Epidemiology – BIPS, Department of Epidemiological Methods and Etiological Research, Bremen, Germany; ^b^ Institute of Statistics, Faculty of Mathematics and Computer Science, Bremen University, Bremen, Germany; ^c^ Department of Food Science, TTZ, Bremerhaven, Germany; ^d^ Unit of Epidemiology and Population Genetics, Institute of Food Sciences, National Research Council, Avellino, Italy

**Keywords:** Children, fungiform papillae, sensory taste perception, bitter, fat

## Abstract

**Background**: To measure sensory taste perception in children with an accurate and reproducible method is challenging and objective measurement methods are scarce.

**Objective**: Aim was to characterize sensory taste perception, by measuring the number of fungiform papillae (FP) and to investigate whether the number of FP is associated with sensitivity for bitter taste and with taste preferences for sweet, salty, fatty or umami in children between 8 and 11 years of age.

**Design**: Number of FP was measured with a digital camera in 83 children in a German subsample of the IDEFICS study. Among those 56 children performed a taste threshold test for bitter and taste preference tests for sweet, salty, fatty and umami. The association between the number of FP and sensory taste perception was analysed.

**Results**: There is a tendency towards a lower number of FP in children with a higher fat preference (30 vs. 25 papillae, p=0.06). Results show no association between the number of FP and neither the bitter taste thresholds nor taste preferences for sweet, salty and umami.

**Conclusion**: Bitter taste threshold might be independent of the number of FP, while the perception of fat was associated with the number of FP.

## Introduction

Dietary behavior is currently considered as one of the factors potentially affecting the development of overweight and obesity, one of the major public health problems among children in Europe. Food choices and dietary behavior in children have been found to be influenced by their taste preferences [1], which differ largely between individuals. Taste preferences might be influenced by individual taste sensitivity [2], which can be examined with sensory taste threshold tests. Conducting these tests with children has been found to be very challenging because the concentration span of children was rather short and their ratings were easily influenced by the examiner [3]. Taste sensitivity has also been evaluated by measuring and counting the number of fungiform papillae (FP). The FP are one of four types of papillae that are located on the tongue and contain multiple taste buds [4]. On top of the FP are taste buds that are responsible for the perception of taste. Although the number of taste buds varies from FP to FP the number of taste buds correlates with number of FP [4]. The number of FP differs between subjects and this may have an impact on the taste sensitivity and subsequently on the preference for the basic tastes [5]. The number of FP can be used as an objective marker that may be less influenced by cognitive abilities of participants. Furthermore, the number of FP cannot be influenced by previously eaten foods and drinks, whereas sensory taste preferences can possibly be influenced by the composition of a previously eaten meal [6–8]. This is an important advantage when using objective measurements: collected data are less probably biased and therefore help to avoid subjective reporting bias (induced through cognitive abilities or by influences of the researcher (e.g. wanting to please the researcher)) and short-term variations in sensory perception. The first method to measure the number of FP in living subjects on a defined area on the tongue by videomicroscopy was established in 1990 by Miller [9]. This extensive method was only suitable for laboratory settings and requires very special and non-portable equipment like a chin holder used for ocular examinations, oral examination chair, tongue chamber and a microscope. Shabake et al. developed a simplified method to measure the number of FP using a special digital camera. This method was evaluated against the formerly established one in a sample of seven adults aged 25–38 years and nine children aged 8–9 years [10]. Both methods provided comparable results. The number of FP was measured in a defined region on the tip of the tongue as this area was more easily accessible than the whole tongue and offered reliable results for the number of FP on the whole tongue [10,11].

The aforementioned challenges of the taste threshold measurements and the lack of alternative feasible methods to measure taste sensitivity in epidemiological studies was an obstacle to obtaining reliable and comparable results from different populations tested in different environments. A robust method in terms of portability that meets these challenges was needed to measure sensory taste sensitivity in children from the general population in different designs in large-scale epidemiological studies.

In the IDEFICS (Identification and prevention of Dietary- and lifestyle-induced health EFects In Children and infantS) study we adapted the method of Shahbake and colleagues to examine the number of FP. The aim of the study was to characterize the sensory taste perception, by measuring the number of fungiform papillae (FP) with a photographic method and further to investigate whether the number of FP is associated with sensitivity for the bitter taste and with taste preferences for sweet, salty, fatty or umami in children between 8 and 11 years of age. If the proposed associations would be observed measuring the number of FP could be a suitable method to objectively measure sensory taste perception in epidemiological field studies of children.

## Methods

### Study design and sample

The measurement of the number of FP was conducted during the follow up in a subsample of 83 children of the German IDEFICS cohort aged 8-11 years [12]. Of the 83 children, 56 participated in the sensory taste perception test module which included the bitter taste threshold as well as the taste preference tests for the sweet, fatty, salty and umami taste. Parents gave their written informed consent on the participation of their children and the children gave their oral consent. Ethical approval was given by the institutional review board of the University of Bremen.

### Instruments

#### Method 1: measuring fungiform papillae

The method to measure the number of FP was developed according to the photographic method described by Shahbake et al. [10].

**Figure 1.**
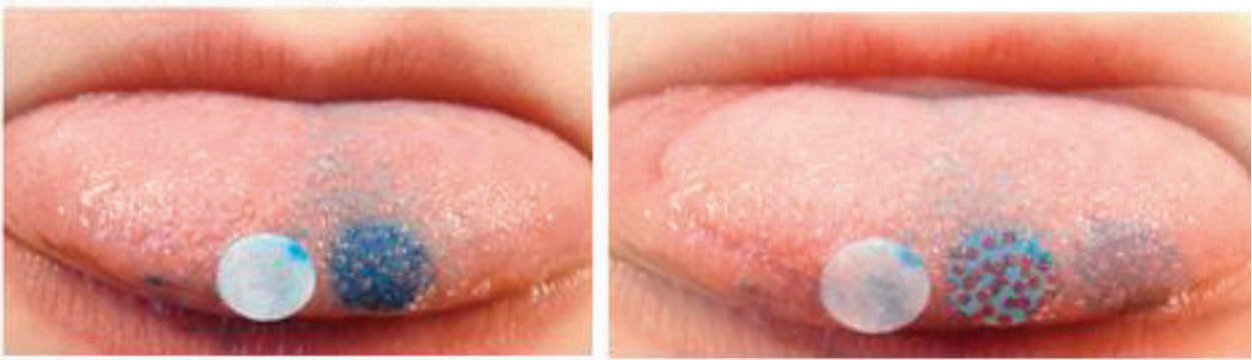
Photos of the stained tongue with and without papillae counted.

Testing was performed in a sitting position and starting with cleansing the mouth by a sip of water. The child placed his or her chin on a non-moving fixture standing on a table in front of them that guaranteed a standard height position during all pictures. The child placed the elbows on the table and fixed the head with the hands. Then the child stretched out the tongue and held it firmly with the lips. The tongue was dried with a filter paper (Whatsmann No. 1). To color the tongue, a blue (brilliant blue E133) colored circled filter paper (6 mm diameter) was placed on the left side of the centerline on the tip of the tongue by using tweezers. The colored filter paper was removed after 5 seconds. An uncolored circled filter paper was placed on the right side of the tongue centerline and was used as a reference circle in case the color on the tongue drifted. Additionally, an individual ID number label was attached to the child’s chin in order to assign the photo to the participant. After removing the filter paper the colored part of the tongue was pictured with a digital camera (Casio Exilim EX-H10, 12,1 megapixel) in macro mode, which was fixed on a tripod. Ten pictures were taken from each child. The pictures were transferred to a computer and were processed as well as analyzed with the program GIMP version 2 (GNU Image Manipulation Programme for Windows) (Figure 1). The number of FP of each participant was counted twice by two independent examiners and the mean number of FP was calculated in order to obtain the number of FP on a circle with a 6 mm diameter.

#### Method 2: sensory taste perception tests

Within the IDEFICS study, two methods were developed to measure (a) taste sensitivity and (b) taste preferences. The development of the standardized test procedures has been previously reported [13].

##### Taste sensitivity test

In the present study we analyzed the bitter taste threshold. A paired comparison staircase test for bitter threshold was applied by testing five aqueous solutions with ascending concentration of caffeine (0.26 to 1.3 mmol l^−1^) against pure water. The first sample of the test solutions that was recognized as being different from water was assumed as the bitter taste threshold, which ranged from 1 to 5 according to the number of solutions.

##### Taste preference tests

To assess the taste preference for sweet, fatty, salty and umami we applied forced-choice paired preference tests with apple juice (sweet) and with crackers (fatty, salty and umami) [13]. Each child had to choose a preferred sample out of a pair. The pairs consisted of two samples, one was the basic sample and the other one a modified sample. Sweet preference was measured with a pair of apple juice. The basic sample contained 0.53% sucrose and the modified sample 3.11% sucrose. Fatty, salt and umami preference were measured with crackers. Basic crackers contained 8% fat, 0.7% sodium chloride and 0% monosodium glutamate and the modified sample 18% fat, 1.6% sodium chloride and 1% monosodium glutamate, respectively. The outcome variables were coded with 1 or 2 depending on the preferred sample of the test pairs.

### Weight, height, BMI *z*-score and weight status

Children’s weight and height was measured in a fasting state using a Tanita BC 420 SMA scale (TANITA, Tokyo, Japan) for weight measurement and a ECA 225 Stadiometer (SECA GmbH & KG, Hamburg, Germany) for height measurement. BMI was calculated and converted to age- and sex-specific *z*-scores [14]. Children were classified into thin/normal weight and overweight/obese using age- and sex-specific cut-points published by Cole et al. [15].

### Statistical analysis

The number of FP that were present in categories of every bitter taste threshold was pictured in a scatterplot to analyze the association of bitter taste threshold and the number of FP. To test whether children with different taste preferences also had a different number of FP, a Mann–Whitney *U*-test was calculated. The same test was applied to test differences of the number of FP between the sexes, weight status and age groups. All analyses were carried out with SAS (Statistical Analysis System, SAS Institute Inc., Cary, USA), version 9.2.

## Results

We analysed the number of FP in 83 children (46 girls and 37 boys; mean age 9.8) of the German IDEFICS study. The characteristics of this subsample were shown in Table 1.

**Table 1.** Characteristics of the study sample (total numbers and percentages).

	Girls	Boys
***N* (%)**	46 (55.4%)	37 (44.6%)
**Age (years)^a^**	9.8 (8.8;10.1)	9.9 (9.4;10.4)
**BMI *z*-score^a,b^**	0.1 (−0.4; 0.9)	0.1 (−0.7; 1.0)
**Overweight/obese^c^**	6 (13.0)	5 (13.5)
**Number of fungiform papillae^a^**	29.3 (23.0; 35.0)	28.5 (24.0; 34.0)
**Bitter taste threshold^d^**		
Threshold 1 (0.26 mmol caffeine/l^a^)	8 (23.5)	3 (13.6)
Threshold 2 (0.51 mmol caffeine/l^a^)	12 (35.3)	5 (22.7)
Threshold 3 (0.77 mmol caffeine/l^a^)	6 (17.7)	4 (18.2)
Threshold 4 (1.03 mmol caffeine/l^a^)	5 (14.7)	1 (4.6)
Threshold 5 (1.29 mmol caffeine/l^a^)	0 (0.0)	3 (13.6)
Non taster	3 (8.8)	6 (27.3)
**Taste preferences^d^**		
High sweet preference^e^	15 (44.1)	9 (39.1)
High salt preference^e^	24 (68.6)	14 (63.6)
High umami preference^e^	14 (40.0)	7 (31.8)
High fat preference^e^	15 (42.9)	6 (27.3)

Abbreviations: BMI *z*-score, Body Mass Index *z*-score.

^a^Numbers are median with interquartile range.

^b^BMI *z*-scores according to Cole et al. [14].

^c^BMI cutoffs according to Cole et al. [15]

**^d^**Sensory tests conducted in a subsample of *n* = 56 children.

^e^Preference for the food sample with the added ingredient over the basic food sample.

**Figure 2.**
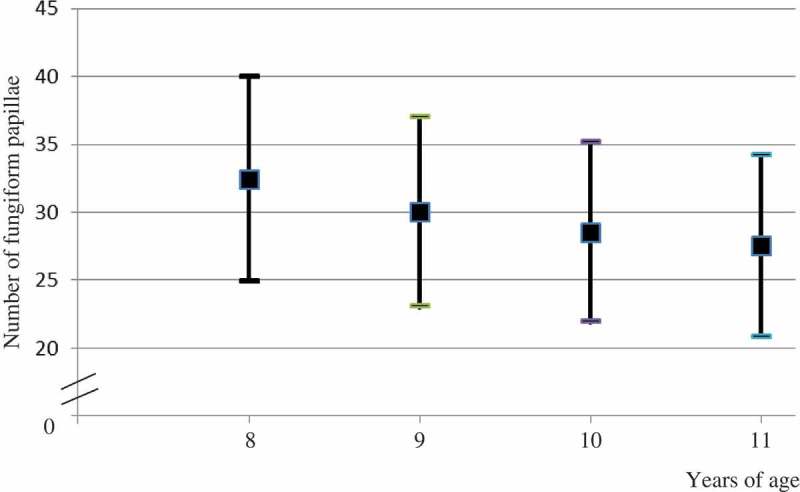
Median (SD) number of fungiform papillae in different age groups.

The median of the number of FP within the defined circle was 29 (interquartile range: 23–35), ranging from 14 to 46. Only 2.4% of the children had less than 15 or more than 40 FP. There were no differences between boys and girls with regard to the number of FP. Normal weight children (*n* = 72) had a mean number of FP of 29.3 (SD = 6.8) and obese/overweight children (*n* = 11) 26.5 (SD = 7.0). The mean number of FP decreased with increasing age (Figure 2).

**Figure 3.**
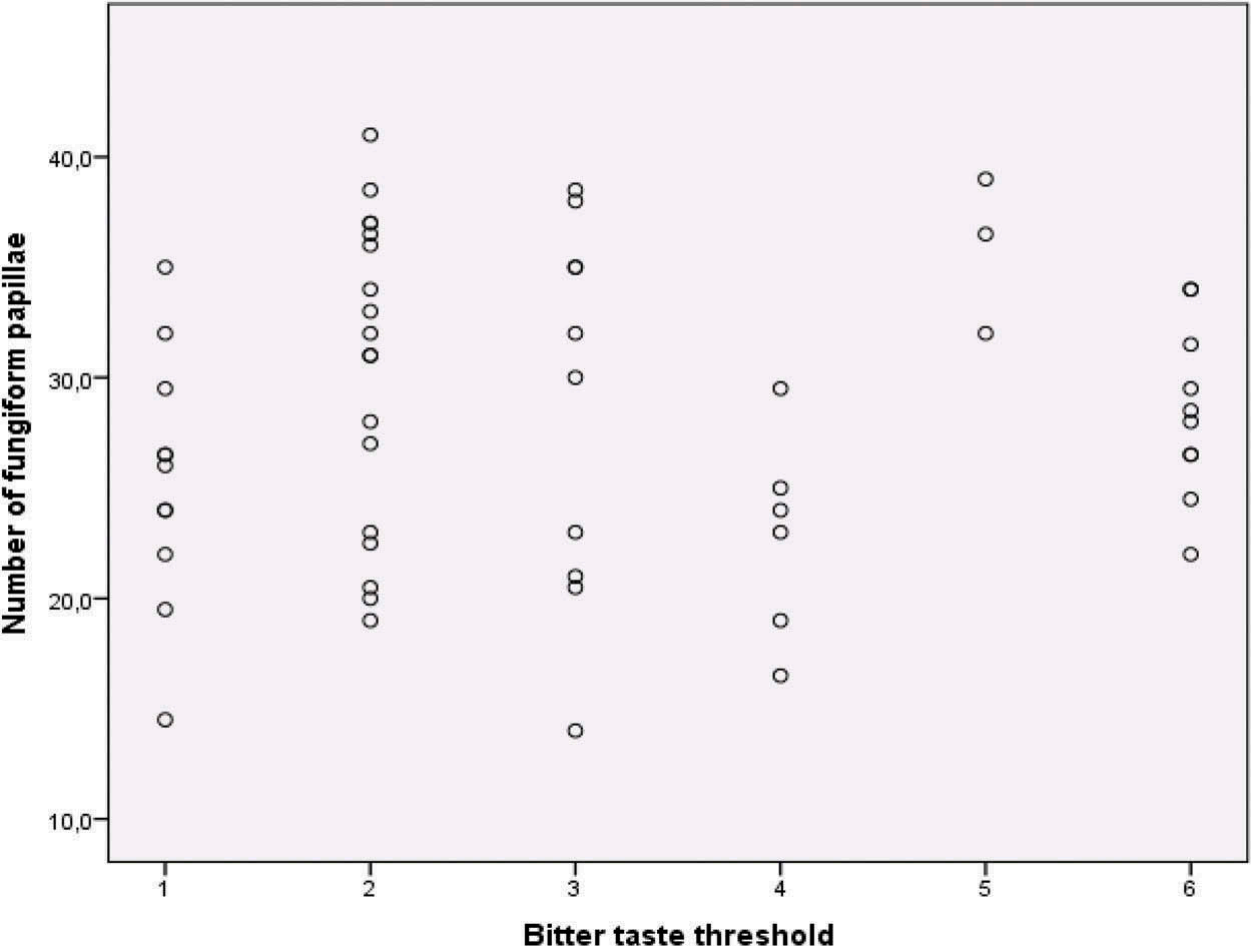
Distribution of fungiform papillae in categories of bitter taste threshold.

There was no association between taste sensitivity for bitter taste and the number of FP. The distribution of the number of FP in categories of the bitter taste threshold is shown in Figure 3.

**Figure 4.**
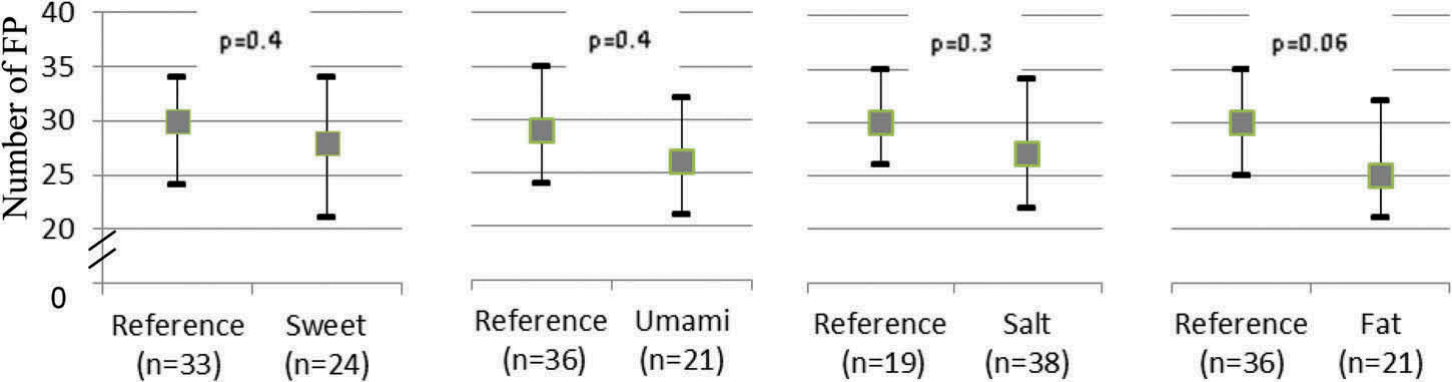
Median (Q1; Q3) number of fungiform papillae in different taste preference groups.

There was no association between the number of FP and taste preferences for sweet, salty and umami. Only regarding fat preference was there a tendency towards a lower number of FP in the group with the higher fat preference (30 vs. 25 papillae, *p* = 0.06) (Figure 4).

## Discussion

We measured the number of FP in 83 children between 8 and 11 years in a free-living setting (during school hours). The median of the number of FP of this sample was 29. This number was higher than that reported in previous studies [10,16]. Due to our limited sample size we conducted descriptive statistics in the present study rather than inductive statistics and no conclusions about associations can be drawn. Interestingly, in our study, it seems that there is a possible decrease of number of FP with every increasing year of age. This could be due to the rapid development of the taste apparatus that occurs during this phase of childhood. The anterior tongue is growing until approximately 8–10 years [17]. Therefore, the number of FP on a defined area decreases during childhood. This could be confirmed with our results. Our result was also in line with Stein et al. and Segovia et al., who showed a higher density of FP in children than in adults [4,18]. We found no differences between the number of FP for boys and girls. Other studies that compared the number of FP in women and men found higher numbers in women [19,20], suggesting that sex differences might occur later in life due to heterogeneous lifestyles, but this needs to be investigated. Two recent studies that investigated FP in children between 7 and 12 and 5- and 17-year-old children found similar numbers of FP on a 6 mm diameter circle [11,21]. The former found a decrease in number of FP with increasing age and also no differences between male and female participants [11]. In our study, we could not confirm an association between weight status and number of FP. Our result may be explained through the overall smaller sample size and by the small number of overweight or obese children.

A smaller subsample of 56 children additionally accomplished the bitter taste threshold test as well as sweet, salty, umami and fat preference tests. Due to the overall study design, sour taste was not investigated in this large-scale epidemiological study across various European countries. These tests were developed for children from the age of six and older. A pre-test was conducted and test–retest reliability was calculated [13]. We analyzed the association between the number of FP and the results of the taste threshold and taste preference tests. Because all taste tests were measured in the whole mouth we believe that a correlation would have been detected between the number of FP on the tip of the tongue with any of these taste perceptions if there was any. We did not find an association between the number of FP and the bitter taste threshold. Previous studies investigated the association between the number of FP and bitter taste perception using suprathreshold concentrations instead of concentrations around the bitter taste threshold as in the present study [16,22]. These previous studies found positive correlations suggesting that the number of FP might be more relevant for the perceived taste intensity than for the taste threshold. The aforementioned studies used propylthiouracil (PROP) to assess the bitter perception. This could have led to a stronger association between bitter perception and the number of FP because the perception of PROP is genetically determined [23]. We used caffeine instead of PROP, as PROP was classified as potentially carcinogenic [24] and was therefore ethically no alternative for use in children. Hall and Hayes et al. described that the thresholds for caffeine correlate with those for PROP and phenylthiocarbamide (PTC), a substance which was used for bitter perception testing before PROP [19,25].

We observed a tendency towards a lower number of FP in children with a higher fat preference. Tepper & Nurse and Nachtsheim & Schlich also investigated the association between the number of FP and fat perception and liking among adults [22,26]. Tepper & Nurse found that participants with more FP could discriminate differences in the fat content of salad dressings whereas participants with fewer FP were not able to do so but preferred the salad dressing with the higher fat content. Nachtsheim & Schlich found that participants with greater number of FP rated the fat content of milk and cream as higher compared to participants with a lower number of FP. The lower preference for crackers with added fat may be explained by the higher fat taste intensity of children with higher number of FP who might therefore need less fat to be satisfied. Consequently, they would choose foods with lower fat content rather than a higher fat content. The lack of significance of the association regarding the fat preference could be due to matrix effects influencing fat release and consequently fat perception. Fat in liquid foods is more ready to diffuse out compared to solid food matrices like crackers. Therefore, liquid foods could be more suitable to measure sensory fat perception.

In the present study we used a digital camera instead of a reflex camera. This could have led to measurement inaccuracies such as misidentification of papillae (counting filiform papillae as fungiform papillae) due to lower resolution of the obtained pictures. A higher resolution would be achievable by using a reflex camera with macro mode and a ring flash.

## Conclusion

We concluded that measuring the number of FP is a suitable method to objectively measure characteristics of taste perception in children, in which objective measurements should be preferred when using a reflex camera with macro mode and ring flash as well as having an appropriate computer program for counting the number of FP. We observed a tendency towards a lower number of FP in children with a higher fat preference. Our results showed no association between the number of FP and the bitter taste thresholds and no association between number of FP and taste preference for sweet, salty and umami.

The issue of using the number of FP as an appropriate marker of taste perception still needs further and more in-depth investigations. In future studies examining children the number of FP should be measured in combination with the perceived taste intensity of different taste qualities.
