# Corrigendum

**DOI:** 10.1080/16546628.2017.1369704

**Published:** 2017-08-28

Jilani H, Ahrens W, Buchecker K, et al. Association between the number of fungiform papillae on the tip of the tongue and sensory taste perception in children. Food Nutr Res. 2017;61:1348865.

https://doi.org/10.1080/16546628.2017.1348865

When the above article was first published online, Wolfgang Ahrens was incorrectly attributed as Wohlfgang Ahrens. This has now been corrected in the online version.

